# Fibrous Dysplasia With Aneurysmal Bone Cyst Presenting as Sinonasal Mass

**DOI:** 10.7759/cureus.24485

**Published:** 2022-04-25

**Authors:** Garima Sharma, Pankaj Sharma, S. Gautham Shankar, Rohit Gupta

**Affiliations:** 1 Department of Radiodiagnosis, All India Institute of Medical Sciences, Rishikesh, Rishikesh, IND

**Keywords:** ground glass matrix, sino-nasal mass, craniofacial disorders, aneurysmal bone cyst, fibrous dysplasia

## Abstract

Craniofacial involvement in fibrous dysplasia is a rare occurrence and complications of it are even rarer. Involvement of the sinonasal region can pose a challenge in both diagnosis and management. We hereby present a case of craniofacial fibrous dysplasia complicated with secondary aneurysmal bone cyst formation in a 15-year-old male patient.

## Introduction

Fibrous dysplasia (FD) is a benign tumor-like disorder of the skeletal system which can affect virtually any bone in the body, representing approximately seven percent of this subgroup [1]. Characterized pathologically by replacement of medullary bone by fibrous tissue, main radiological features include osseous expansion and ground glass appearance. Of its four subtypes, craniofacial fibrous dysplasia can present as polyostic (50%) or monostic (25%) variants [2].

Complications are not uncommon in FD since the affected bones are rendered weak and prone to pathological fractures. Aneurysmal bone cysts (ABC) are rare benign tumor-like lesions of vascular origin affecting typically involve the long bones of the extremities [3], membranous bones of the thorax and pelvis, or vertebrae. It can be primary or secondary to any pre-existing pathology like giant cell tumor, chondromyxoid fibroma, or fibrous dysplasia. We present a case of a 15-year-old male patient with craniofacial FD complicated by the development of ABC, leading to a rapidly developing sino-nasal mass.

## Case presentation

A 15-year-old male patient, previously diagnosed with craniofacial FD, presented with a rapidly developing mass lesion involving the nasal cavity with proptosis of the left eye. There was no history of epistaxis. Clinical examination revealed facial dysmorphism and proptosis of the left eye. Endoscopic examination of the nasal cavity shows a friable reddish mass lesion involving the bilateral nasal cavity. Clinical differentials included vascular mass lesions, i.e, hemangioma or juvenile nasopharyngeal angiofibroma. The patient was advised of Magnetic Resonance Imaging (MRI) for further characterization.

**Figure 1:**
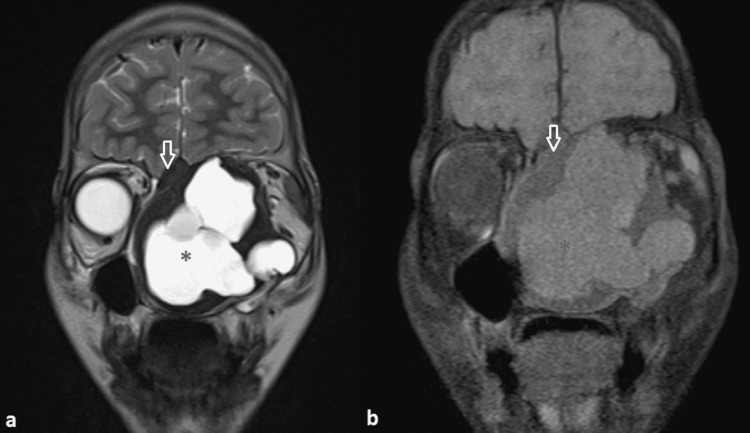
Coronal MRI images (a) T2 weighted image and (b) T1 weighted MRI image A lytic expansile lesion is seen involving the bilateral nasal cavity with a central cystic T1/T2 hyperintense (black asterisk) component and peripheral T1/T2 hypointense regions (white open arrow). MRI - Magnetic Resonance Imaging

On MRI, a large multi-lobulated lytic expansile lesion with peripheral T1/T2 hypointense component was seen involving the nasal cavity causing its expansion (Figure 1).

**Figure 2:**
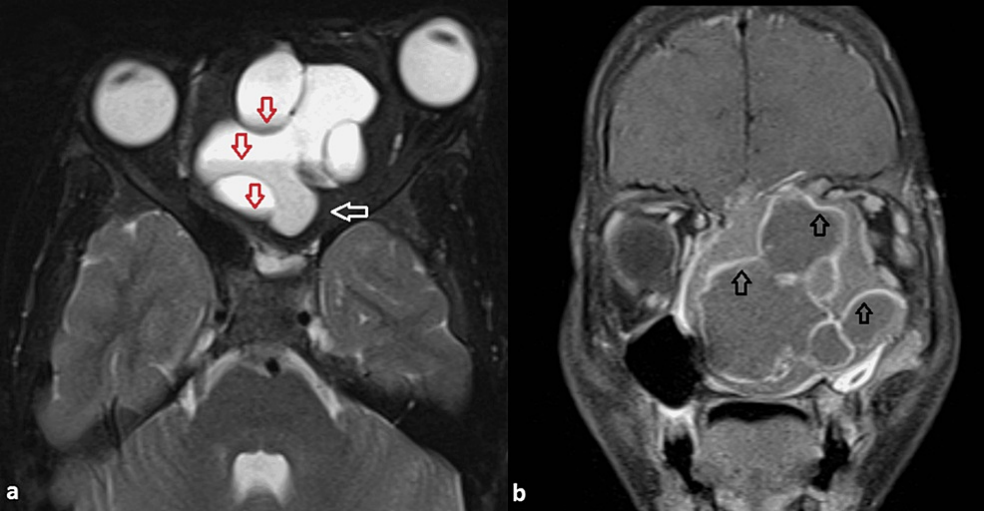
(a) Axial T2 weighted MRI image and (b) Coronal T1 fat-suppressed post-contrast image Fluid-fluid levels are seen within the cystic component of the lesion (indicated by red open arrows in 2a). The periphery of the lesion shows the peripheral T2 hypointense solid component (white open arrow in 2a). Proptosis of the left eye is seen. In the post-contrast image, peripheral enhancement is seen in cystic as well as solid components of the lesion (open black arrows in 2b). MRI - Magnetic Resonance Imaging

Nasal turbinates and nasal septum were not visualized. Fluid-fluid levels were seen in the cystic component. The lesion was extending and involved the ethmoid and sphenoid sinus. Mass effect was seen over bilateral orbits with proptosis of the left eye and hard palate, however, no intra-orbital or intra-oral extension was seen (Figure 2). Contrast angiography sequences showed no major arterial feeder in the location of the lesion. 

**Figure 3:**
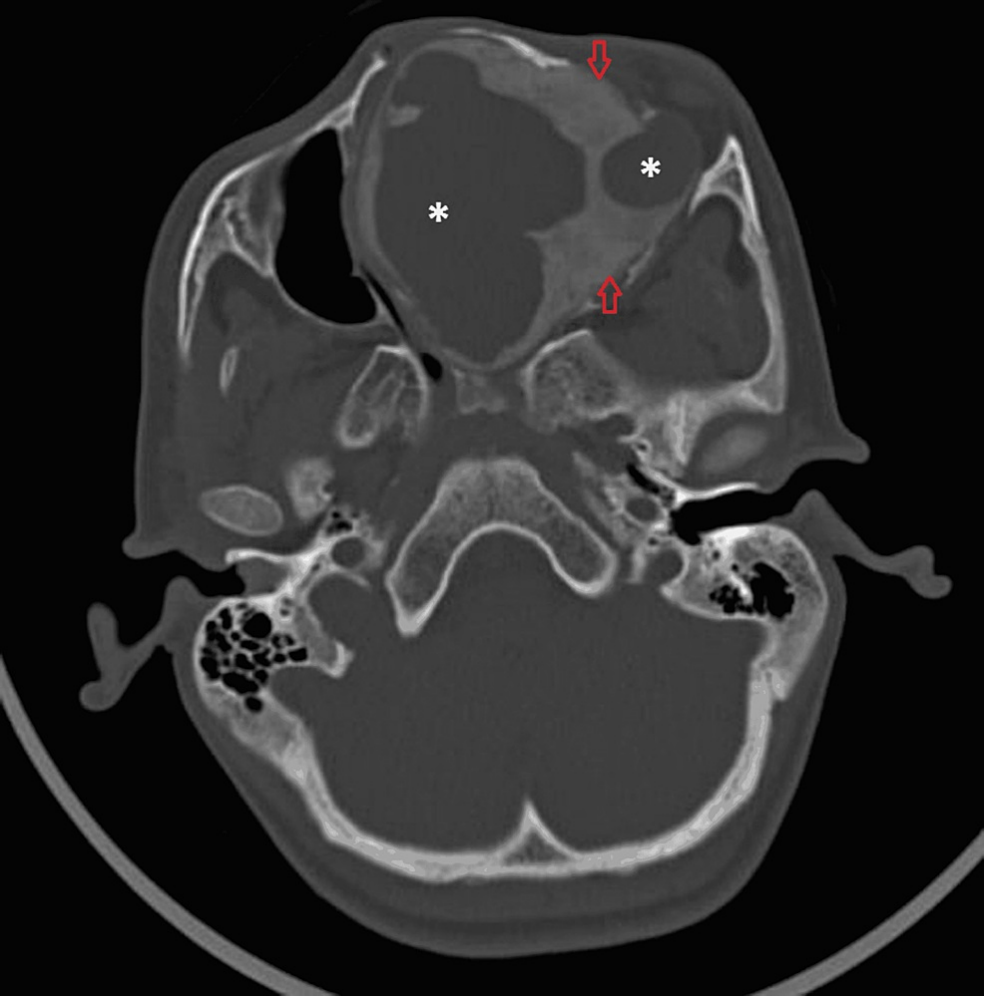
Axial CT image in bone window A lytic expansile lesion involving the nasal cavity central cystic region (white asterisk) and peripheral bone showing ground glass matrix (open red arrows). CT - Computed Tomography

On computed tomography, the peripherally involved bone showed a ground-glass matrix with the cortical breach at a few places (Figure 3).

Incisional biopsy from the involved bone was suggestive of a fibro-osseous lesion. A combined high transcranial and transfacial approach was utilized. Intra-operatively, a large cystic lesion filled with dark hemorrhagic fluid was seen occupying the nasal cavity. The bone present at the margin of the lesion was thickened and was of gritty consistency. En-bloc resection including the involved bone was done. Adequate bone clearance was achieved at all margins except posteriorly and superiorly in the region of the left cribriform plate as it was significantly thinned out. There were significant cosmetic and visual improvements after surgery. 

The surgical specimen showed two components within. The solid component at the periphery of the lesion showed scattered immature bone trabeculae within the fibrous matrix, characteristic of FD. The cystic component of the lesion showed blood-filled spaces with scattered multinucleated giant cells suggestive of ABC. 

## Discussion

Sino-nasal mass range from benign etiologies such as antrochoanal polyps and papillomas to malignant lesions such as juvenile nasopharyngeal angiofibroma (JNA) and lymphoma. A complete imaging workup is necessary not only to delineate and characterize the primary pathology but also the complications. Radiographs of the skull and paranasal sinuses can be initially done, however, does not provide much information. CT is useful for the assessment of the structure of affected bone and complications like pathological fractures, while MRI is an excellent modality for the assessment of intraorbital or intracranial extensions of the lesion. Most benign etiologies such as polyps are solid lesions with associated features of sinusitis,i.e., mucosal thickening and thickening of sinus walls. While the malignant masses are most destructive, invading various surrounding structures. Vascular tumors like JNA will show avid enhancement with multiple arterial feeders identified on angiography. Cystic nasal masses in children include congenital entities such as congenital nasolacrimal duct mucocele, dermoid cyst, and encephalocele, inflammatory and infectious lesions such as mucoceles and polyps, benign or malignant neoplasms with internal areas of degeneration and necrosis. Multiloculated cystic lesion with fluid-fluid levels is a characteristic imaging feature of ABC which was the clincher in our case. Further, the assessment of surrounding structures such as nerves or bones can provide a clue to the origin of the lesion. In our case, the surrounding bone showed expansion with the ground glass matrix suggestive of FD.

FD can often be complicated by pathological fractures. Secondary development of ABC in FD is a rare occurrence, with only 15 cases reported so far [4-8]. Of these, three occurred in the sino-nasal region. The etiopathology of the development of ABC is postulated to be due to local hemodynamics disturbances with bone resorption or hemorrhage secondary to pathological fracture. Hence, it is often seen in bones primarily affected by other pathology. Multiple reports of secondary development of the ABC from a pre-existing lesion such as in benign tumors like a unicameral bone cyst, giant cell tumor, osteoblastoma, chondromyxoid fibroma, as well as malignant tumors like osteosarcoma have been described.

The concomitant occurrence of FD and ABC was first described by Branch et al. [4] in 1986. Składzień et al. [9] reported a case of giant ABC associated with FD involving both nasal cavities, encroaching upon para-nasal sinuses and both orbits, with skull base destruction in the anterior cranial fossa. Clinical presentation in complicated craniofacial FD depends on the location and rapidity of the growth of the lesion. Involvement of the sino-nasal region may present as nasal blockage, recurrent sinusitis, epistaxis, headache, proptosis, or visual loss. Intra-cranial extension and rupture can even present as subarachnoid hemorrhage [10].

En-bloc resection with bone grafting is the treatment of choice in most cases [11]. Complex anatomical sites with proximity to vital structures and risk of significant postoperative morbidity may preclude a complete en-bloc resection, however, the aim is to remove the greatest possible amount of diseased bone without affecting the functionality of adjacent vital structures. Pre-operative embolization for tumor vascularity reduction and blood-less surgical field is sometimes done [6]. As no significant vascularity or any major arterial feeders were identified on magnetic resonance (MR) angiography, pre-operative embolization was not attempted in our case. Various other therapies like adjuvant radiotherapy, sclerotherapy, high-speed burr, argon beam coagulation, cryotherapy, and even medical management with drugs like denosumab are currently under study [12].

## Conclusions

This case represents a common condition with an uncommon complication and clinical presentation. Sino-nasal mass in a young patient can have several differential diagnoses, ranging from congenital, inflammatory, and infective etiologies to neoplastic processes. A pre-treatment cross-sectional imaging can adequately characterize and delineate these lesions as well as guide the treatment options. Adequate anatomical knowledge with a mind open to the possibility of lesions arising from different tissues, i.e, sino-nasal mucosa, bony structures, respiratory epithelium, vessels, and lymphatics, present in this region is necessary to evaluate masses of this complex anatomical site. Also, assessment of lesions as well as surrounding structures is necessary for guiding the pre-operative and operative management of the patient.
